# Outstanding Separation Performance of Oil-in-Water Emulsions with TiO_2_/CNT Nanocomposite-Modified PVDF Membranes

**DOI:** 10.3390/membranes13020209

**Published:** 2023-02-08

**Authors:** Laura Fekete, Ákos Ferenc Fazekas, Cecilia Hodúr, Zsuzsanna László, Áron Ágoston, László Janovák, Tamás Gyulavári, Zsolt Pap, Klara Hernadi, Gábor Veréb

**Affiliations:** 1 Department of Biosystem Engineering, Faculty of Engineering, University of Szeged, Moszkvai Blvd. 9., H-6725 Szeged, Hungary; 2 Department of Physical Chemistry and Materials Science, University of Szeged, Rerrich Béla Sqr. 1, H-6720 Szeged, Hungary; 3 Department of Applied and Environmental Chemistry, Institute of Chemistry, University of Szeged, Rerrich Béla sq. 1, H-6720 Szeged, Hungary; 4 Institute of Physical Metallurgy, Metal Forming and Nanotechnology, University of Miskolc, Miskolc-Egyetemváros, C/1 108, H-3515 Miskolc, Hungary

**Keywords:** membrane filtration, oil emulsion, nanocomposite, TiO_2_, carbon nanotube, MWCNT, PVDF, enhanced flux, reduced resistance, pressure-dependent effects

## Abstract

Membrane filtration is an effective technique for separating micro- and nano-sized oil droplets from harmful oil-contaminated waters produced by numerous industrial activities. However, significant flux reduction discourages the extensive application of this technology; therefore, developing antifouling membranes is necessary. For this purpose, various titanium dioxide/carbon nanotube (TiO_2_/CNT) nanocomposites (containing 1, 2, and 5 wt.% multi-walled CNTs) were used for the modification of polyvinylidene fluoride (PVDF) ultrafilter (250 kDa) membrane surfaces. The effects of surface modifications were compared in relation to the flux, the filtration resistance, the flux recovery ratio, and the purification efficiency. TiO_2_/CNT_2%_ composite modification reduced both irreversible and total filtration resistances the most during the filtration of 100 ppm oil emulsions. The fluxes were approximately 4–7 times higher compared to the unmodified PVDF membrane, depending on the used transmembrane pressure (510, 900, and 1340 L/m^2^h fluxes were measured at 0.1, 0.2, and 0.3 MPa pressures, respectively). Moreover, the flux recovery ratio (up to 68%) and the purification efficiency (95.1–99.8%) were also significantly higher because of the surface modification, and the beneficial effects were more dominant at higher transmembrane pressures. TiO_2_/CNT_2%_ nanocomposites are promising to be applied to modify membranes used for oil–water separation and achieve outstanding flux, cleanability, and purification efficiency.

## 1. Introduction

A primary challenge of the 21st century is to ensure suitable freshwater of sufficient quality and volume for the continuously growing population, agriculture, and industry, as significant parts of the world are already struggling with water scarcity [1,2,3]. For this purpose, it is crucial to develop our wastewater treatment technologies. Beyond environmental protection reasons, economic concerns (such as increasing costs of processing water and wastewater discharge) also urge industries to use advanced wastewater treatment methods. Such methods could provide higher purification efficiency, the avoidance of penalties, or even the support of circular economies by water recirculation, which will inevitably be necessary as industrial water utilization is predicted to increase by 400% by 2050 [4,5].

Several industries produce harmful wastewater, one of which is the group of oil-contaminated waters. There is an urgent need for more efficient and cost-effective treatment methods for these wastewaters, since they are produced in high quantities (by several activities, such as crude oil extraction, oil refining, production of lubricants, metalworking, and cleaning of vehicles) and oily contaminants contain several toxic or even carcinogenic compounds (such as hydrocarbons, heavy metals, and phenols); therefore, they pose a serious risk to the environment and human health [6,7,8].

The modern techniques for the purification of oily wastewaters are membrane separation, biological treatment, and advanced oxidation processes [9,10,11]. Membrane filtration has several unique properties, such as high separation efficiency, small footprint, facile operation, and good integrability. Therefore, it could serve as a promising supplementary technique to low-cost traditional methods, such as chemical destabilization supported sand filtration, flotation, and skimming [9,10,11,12,13], if the problem of the oil-caused quick and significant fouling could be solved [7,9,14,15], since fouling reduces not only water permeability and often purification efficiency, but also the lifespan of membranes while it increases operational costs [14].

Membrane fouling can be reduced by the reduction of the interaction between oily foulants and the membrane surface. This can be carried out by increasing the hydrophilicity via methods such as sulfonation, carboxylation, UV-induced grafting, and nanoparticle-based surface modifications [6,14,15,16].

Several nanoparticles have been investigated for the fabrication of antifouling membranes, including carbon-, silver-, silica-, aluminum-, titanium-, and magnesium-based nanoparticles [15,17]. Nanomaterial-modified membranes could result in improved physicochemical properties, increased permeability, less fouling, or better performance over a wider temperature and pH range [15,16,17,18].

Among the nanoparticles, titanium dioxide (TiO_2_) is one of the most investigated materials [6,18,19,20,21,22,23,24,25,26,27,28,29] due to its hydrophilic nature, chemical stability, availability, and photocatalytic activity (which provides the possibility of preparing self-cleaning membrane surfaces). The beneficial effects of TiO_2_-based membrane modification have been proved in relation to both surface coating [18,19,21,22,23,25] and blending [17,20,28,29] methods. Carbon nanotubes (CNTs) also have a growing interest in the field of advanced membranes, which exhibit high flux, selectivity, and low-fouling properties even against biofouling [30,31]. The combined use of TiO_2_ and CNTs as nanocomposites can offer further advantages, such as enhanced photocatalytic activity due to the reduction of electron/hole recombination [32,33,34]. Moreover, their combined use for membrane surface modification has already been proven beneficial in some studies, resulting in better self-cleaning properties, higher fluxes, and lower fouling tendency [6,23,27,35].

The TiO_2_/CNT ratio of nanocomposite-coated membranes most probably affects the advantages in relation to the filtration properties of crude oil-contaminated waters. However, this issue has not been investigated in detail to the best of our knowledge. Therefore, in the present study, 1–5 wt.% CNT-containing TiO_2_/CNT nanocomposites were used to modify polyvinylidene fluoride (PVDF) ultrafilter membranes. The membranes were used to separate 100 mg/L crude oil-containing oil-in-water emulsions to investigate the effects of CNT content on the flux, the filtration resistance, the flux recovery ratio, and the purification efficiency.

Additionally, the effects of the applied transmembrane pressure on these parameters were also investigated for the most beneficial composite-modified and unmodified commercial PVDF membranes. This way, the pressure dependence of the achievable advantages resulting from the nanocomposite-based surface modification was investigated. To the best of our knowledge, this aspect of TiO_2_/CNT composite-modified membranes has also not been investigated in relation to oil emulsions. The main aim of the present research is to contribute to the development of membrane surfaces, which prevents the adherence of oily contaminants even at high transmembrane pressures. Such membranes could be used to reduce the environmental damage caused by oil pollution.

## 2. Materials and Methods

### 2.1. Nanomaterial-Based Modification of the Membrane Surfaces

The commercial PVDF membranes were modified with TiO_2_ (Aeroxide P25, Evonik Industries, Essen, Germany; d = 25–39 nm) and with 1, 2, or 5 wt.% multi-walled CNTs (Nanothinx NTX1, Patra, Greece; l ≥ 10 μm; d = 15–35 nm) containing TiO_2_/CNT composites. Total weights of 40 mg from the nanomaterial(s) were suspended in 100 mL volumes of 2-propanol (c = 400 mg/L) and dispersed for 2 min with a high-power (200 W, 24 kHz, amplitude = 1, cycle = 100%) ultrasonic homogenization (Hielscher UP200S, Teltow, Germany) combined with intense magnetic stirring. Then, the TiO_2_ nanoparticles or the TiO_2_/CNT composites were immobilized on the commercial PVDF membranes (New Logic Research Inc., Minden, Louisiana, USA; 250 kDa; ~1.0 mg/cm^2^ catalyst coverage) by physical deposition: the suspension was filtered through the membrane in a batch-stirred membrane reactor (Millipore, XFUF07601, Burlington, Massachusetts, USA), using 0.3 MPa transmembrane pressure; then, the membranes were dried in the air at room temperature.

### 2.2. Characterization of the Membrane Surfaces

Contact angle measurements (Dataphysics Contact Angle System OCA15Pro, Filderstadt, Germany) were carried out to characterize the hydrophilicity of the membrane surfaces. A 10 µL volume of ultrapure water was carefully dropped onto the surfaces, and contact angles formed between the given membrane and the ultrapure water droplet were measured immediately (within 1 s). The measurements were repeated four times, and average values were calculated.

The zeta potential values of some membrane surfaces were also measured using a streaming potential technique in an Anton Paar SurPASS 3 device (AntonPaar Gmbh, Graz, Austria) equipped with an adjustable gap cell. During a measurement, two pieces of the membranes (10 mm × 20 mm) were fixed with double-sided adhesive tapes. The measurements were performed at a pH range of ~2–8 (c_KCl_ = 0.001 mol/L), which was adjusted by adding HCl and KOH solutions. The system was equipped with a pH electrode, which continuously monitored the pH value.

### 2.3. Production of the Model Oily Wastewater

Crude oil (MOL Zrt., Algyő, Hungary) containing oil-in-water emulsion was prepared in two steps: 1 wt.% crude oil was added to ultrapure water (Elga-Veiola, PureLab chorus, High Wycombe, UK) and stirred intensely (Skil F0151415AC, Teuchern, Germany) at a stirring speed of 35,000 rpm for 30 s. Then, an additional 10 min of high-power (200 W, 24 kHz, amplitude = 1, cycle = 100%) ultrasonic fragmentation (Hielscher UP200S, Teltow, Germany) was applied for the diluted dispersion (T = 25 °C), to obtain a stable oil-in-water emulsion (c_oil_ = 100 mg/L; d_droplets_ = 0.1–1.5 µm).

### 2.4. Membrane Filtration of the Oil Emulsions

The as-prepared oil emulsions were membrane-filtered in a batch-stirred membrane reactor (Millipore XFUF07601, Burlington, Massachusetts, USA) equipped with the given membrane (active filtration area = 36.2 cm^2^), using a 0.1 MPa transmembrane pressure and a 5.83 s^−1^ stirring speed (350 rpm). In a series of experiments, the transmembrane pressure was increased from 0.1 to 0.2 and 0.3 MPa. In all filtration experiments, 250 mL emulsion was filtered, until a volume reduction ratio (VRR) of five was reached (until 200 mL permeate production). Purification efficiencies were characterized by turbidity, chemical oxygen demand, and extractable oil content measurements.

### 2.5. Calculation of the Flux, the Filtration Resistances, and the Flux Recovery Ratio

The fluxes (*J*) were recorded with a computer-controlled scale (Kern EG420-3NM, Balingen, Germany) and calculated as follows:

(1) $$J=\frac{∆V}{A\cdot ∆t}\cdot 100\%\;(\frac{L}{m^{2}h})$$

where *J* is the permeation flux, Δ*V* is the permeate volume (*L*), *A* is the effective membrane area (m^2^), and Δ*t* is the time of the given interval (h).

The membrane resistance (*R_M_*) was calculated as follows:

(2) $$R_{M}=\frac{∆p}{J_{W}\cdot \eta_{W}}\;\left(m^{-1}\right)$$

where Δ*p* is the applied transmembrane pressure (Pa), *J_W_* is the flux of pure water on the clean membrane (L/m^2^·h), and *η_W_* is the viscosity of the water (Pa·s).

The irreversible resistance (*R_Irrev_*) was determined by measuring the water flux on the used membrane after the filtration of the emulsion, followed by a purification step (intensive rinsing with distilled water):

(3) $$R_{Irrev}=\frac{∆p}{J_{WA}\cdot \eta_{W}}-R_{M}\;\left(m^{-1}\right)$$

where *J_WA_* is the water flux after the cleaning procedure.

The reversible resistance (*R_Rev_*) can be calculated as follows:

(4) $$R_{Rev}=\frac{∆p}{J_{c}\cdot \eta_{WW}}-R_{Irrev}-R_{M}\;\left(m^{-1}\right)$$

where *J_c_* is the flux at the end of the filtration and *η_ww_* is the viscosity of the emulsion. 

The total resistance (*R_T_*) can be calculated as the sum of the previous three resistances:

(5) $$R_{Rev}=R_{M}+R_{Irrev}+R_{Rev}\;\left(m^{-1}\right)$$

The flux recovery ratios (*FRRs*) were calculated from the pure water fluxes, measured before the filtration of the emulsion (*J*_0_) and after the oil filtration and the purification of the membranes (*J*), as follows:

(6) $$FRR=\frac{J}{J_{0}}\cdot 100\%\;\left(\%\right)$$

### 2.6. Characterization of the Purification Efficiencies

Purification efficiencies were characterized by measuring the turbidity, the chemical oxygen demand (COD), and the extractable oil content of the oil emulsions and the permeates.

Turbidity values (NTU: Nephelometric Turbidity unit) were measured with a Hach 2100 N-type turbidity meter (HACH LANGE GmbH, Düsseldorf, Germany).

The COD was measured by the potassium dichromate oxidation method, using standard test tubes (Hanna Instruments, Szeged, Hungary), a Lovibond ET 108 (Dortmund, Germany) digester (t = 2 h, T = 150 °C), and a Lovibond COD Vario (Dortmund, Germany) spectrophotometer.

The extractable oil content (TOG/TPH concentration) was measured with a Wilks InfraCal TOG/TPH analyzer (Wilks Enterprise Inc., Norwalk, Connecticut, USA), using hexane for the extraction. The purification efficiency (*R*) was determined as follows:

(7) $$R=\left(1-\frac{a}{a_{0}}\right)\cdot 100\%\;\left(\%\right)$$

where *a*_0_ is the turbidity, COD, or TOG/TPH value of the feed, while *a* indicates the turbidity, COD, or TOG/TPH value of the permeate.

## 3. Results and Discussion

### 3.1. Effects of Surface Modifications on Fluxes and Filtration Resistances at a 0.1 MPa Transmembrane Pressure

**Figure 1:**
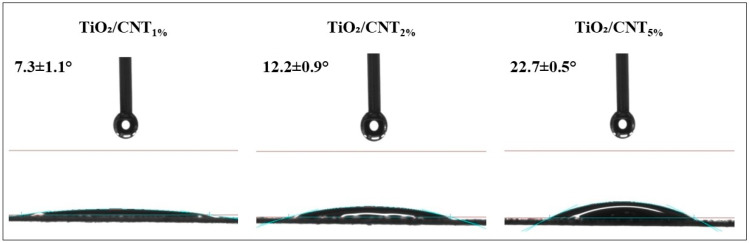
Contact angles formed between the given titanium dioxide/carbon nanotube (TiO_2_/CNT) nanocomposite-coated membrane surface and the ultrapure water droplet (10 µL), measured immediately (within 1 s) after dropping.

The permeability of the PVDF membrane significantly decreased due to the different surface modifications, which indicates the presence of compact nanomaterial-based coatings on the membrane surfaces. The commercial PVDF membrane provided a 3080 L/m^2^h water flux, while the pure (100%) TiO_2_ coating reduced the flux to almost one-third (1130 L/m^2^h). Parallelly with the increase of CNT content (1%, 2%, and 5%) of the nanolayer, the flux values showed a further decrease (1110, 860, and 700 L/m^2^h, respectively). This can be associated with the hydrophobic nature of CNTs, which can be more significant at higher CNT contents (Figure 1).

**Figure 2:**
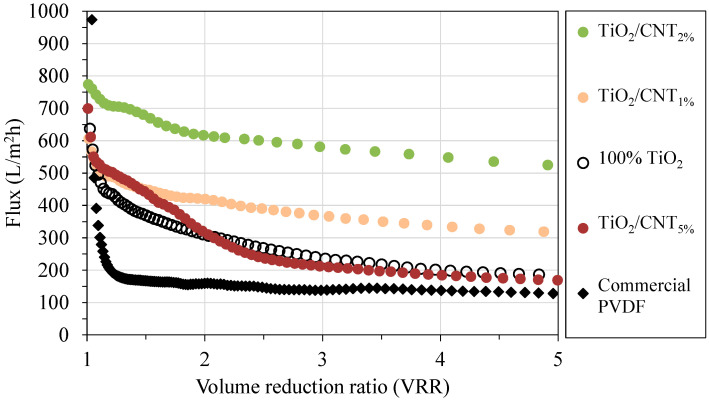
Flux values recorded during the filtration of oil emulsions at a 0.1 MPa transmembrane pressure.

The representative flux curves—recorded during the filtration of the oil emulsions with the different membranes—are presented in Figure 2. The flux measured for the commercial PVDF membrane was the lowest by far. Despite the high initial values, the flux decreased intensively from the very beginning of the filtration: at a VRR of 1.2, it already decreased below 200 L/m^2^h, which further decreased to 130 L/m^2^h by the end of the filtration (VRR = 5). The shape of this flux curve represents the main problem of the membrane filtration of oil emulsions very well, which is the immediate decrease of the flux due to the rapid build-up of the hydrophobic oil layer and the pore fouling caused by easily deformable oil droplets [36].

**Figure 3:**
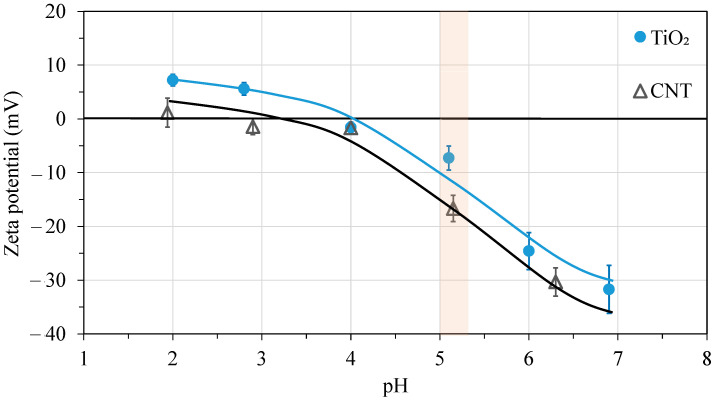
Zeta potentials of CNT and TiO_2_ coatings (the pH of the oil emulsion was 5.2 ± 0.15).

In accordance with the literature [18,20,23,25,28], a significantly higher flux value (185 L/m^2^h at VRR = 5) was achieved for the pure TiO_2_-coated membrane, while TiO_2_/CNT composite modifications (at lower CNT concentrations: 1% and 2%) were more beneficial by providing ~2.4 times higher (310 L/m^2^h) and almost 4 times higher (510 L/m^2^h) fluxes than the commercial membrane, respectively. These positive effects on fluxes can be associated with the more negative surface zeta potential of the carbon nanotube (compared to that of pure TiO_2_; Figure 3). This could result in an increased electrostatic repulsive force between the negatively charged oil droplets (–9.6 ± 3 mV) and the negatively charged membrane surfaces at the pH value of the oil emulsion (pH = 5.2 ± 0.15). The repulsive force could reduce the adherence of the oil droplets to the membrane surface and, therefore, the formation of the hydrophobic cake layer on the surface.

The interpretation of the results described above is in accordance with the findings of some other researchers. Zhang et al. [28] also concluded that the more negative zeta potential (achieved by the PEG/TiO_2_ modification of a PVDF membrane) contributed to the improved antifouling property. Chen et al. [37] also reported reduced fouling for silicon carbide-modified ceramic membranes (with more negative zeta potentials) used to separate oil-in-water emulsions. Furthermore, in an earlier study [38], the pre-ozonation of an oil-in-water emulsion also significantly enhanced the flux via the significantly more negative zeta potential of the emulsion. Regarding CNT addition, the publication of Esfahani et al. [39] can be mentioned, in which the authors reported that the charge of the membrane surface became more negative due to the presence of CNTs, and this resulted in reduced fouling during the filtration of BSA solution (in this study, the oil emulsion was not investigated). Moreover, Moslehyani et al. [31] oxidized the surface of CNTs to achieve more negative zeta potentials and lower fouling with surface-modified CNT-containing membranes (during the separation of oil-in-water emulsions).

The composite containing 5% CNTs resulted in a flux of 170 L/m^2^h at the end of the experiment (VRR = 5), similar to that obtained for the membrane modified with pure TiO_2_ (185 L/m^2^h), even though the shape of the flux curve (Figure 2) indicates moderate fouling at the beginning of the filtration (VRR = 1–2). The significant CNT content of the composite could reduce the hydrophilic nature of the surface (Figure 1), which resulted in higher resistance to the water flow. It could also help the formation of the oily cake layer on the surface, slowing the filtration more intensely. These observations are in line with the findings of Hudaib et al. [40], who also reported reduced water permeability and lower flux (during the filtration of oil emulsions) above a specific CNT concentration (>0.05–0.1 wt. % CNT content in their PVDF/MWCNT/polypyrrole mixed matrix membranes).

**Figure 4:**
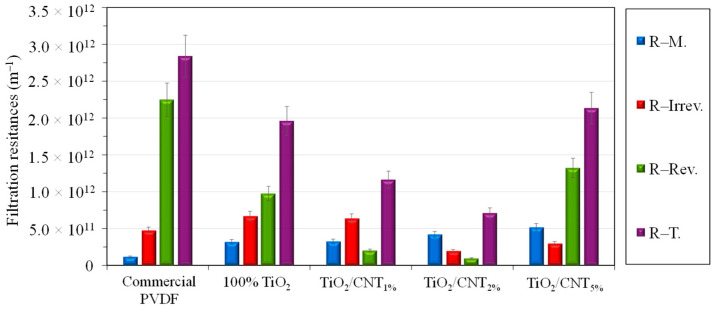
Calculated filtration resistances (when a transmembrane pressure of 0.1 MPa was applied).

For further discussion, the filtration resistances were also calculated (Figure 4), including the own resistance of the membranes (R–M.), the reversible resistance (R–Rev.; caused by the contaminant layer that can be rinsed off the surface and by the concentration polarization layer), the irreversible resistance (R–Irrev.; resulting from the permanently adhered contaminants of the surface and by the fouling of the pores), and the total filtration resistance (R–T.; the sum of the previous three resistances).

The lowest membrane resistance (R-M.) was calculated for the unmodified PVDF membrane (in accordance with its highest pure water flux), which significantly increased after coating with TiO_2_, and further increased with increasing CNT contents.

All the investigated membrane modifications reduced the total filtration resistance, mainly through the significant reduction of the reversible resistance. The lowest total filtration resistance was calculated for the TiO_2_/CNT_2%_-coated membrane, which resulted in a significant reduction of both the irreversible and reversible resistances (the latter was negligible), which were lower than the own resistance of the membrane. These findings prove the significantly inhibited adhesion ability of the oily contaminants on this membrane surface. This composite composition could provide the most beneficial effect, due to the significantly increased hydrophilicity of the membrane and the more negative zeta potential in the presence of CNTs, since the adhesion of oil droplets is determined primarily by electrostatic interactions (influenced by the zeta potential) and van der Waals interactions (influenced by the hydrophilic/hydrophobic nature of the membrane surface), according to the Dejaguin–Landau–Verwey–Overbeek (DLVO) theory [41] (naturally other characteristics, such as roughness, porosity, and other structural properties, are also affecting factors).

Since the interactions between colloidal oil droplets and membranes can be determined by the resultant of van der Waals and electrostatic forces, the changes in TiO_2_/CNT ratio have opposite effects. This is because TiO_2_ nanoparticles are very hydrophilic and although the surface of CNTs has a slightly more negative zeta potential, it is still very hydrophobic. On the one hand, the TiO_2_/CNT_1%_ and the pure (100%) TiO_2_ coatings resulted in slightly more hydrophilic surfaces than the TiO_2_/CNT_2%_ coating (Figure 1), but the 1% CNT content may not have been enough to increase the electrostatic repulsive forces between the composite coating and the oil droplets. On the other hand, the significantly lower hydrophilicity (higher contact angle; Figure 1) of the 5% CNT-containing composite coating, which contributed to the highest membrane resistance and the lowest pure water flux in the series, could overcompensate the beneficial effect originating from the presence of negatively charged CNTs. The shape of the flux curve measured for this membrane (Figure 2) also indicates that a 5% CNT content could inhibit the adhesion of oil droplets only at the beginning of the filtration (VRR = 1–2). After reaching a VRR of 2, the concentrated oily contaminants attached intensely onto the surface, resulting in high reversible and total filtration resistances (Figure 4), and ultimately a slightly lower flux than the flux measured for the membrane with a pure TiO_2_ coating (Figure 2). Regarding this, the recent findings of Hudaib et al. [40] can be mentioned again, who reported decreasing total filtration resistances for PVDF/MWCNT/polypyrrole membranes with 0.0025%, 0.05%, and 0.1% CNT contents during the filtration of synthesized refinery wastewater, but at a 0.3% CNT content, a higher total filtration resistance was observed. Thus, in the mentioned study, the 0.1% CNT content was the optimal value, but the membranes did not contain TiO_2_, and the flux did not exceed 100 L/m^2^h during the filtration of the emulsion.

Considering the results obtained in this study so far, the following experiments were carried out using the TiO_2_/CNT_2%_ composite-modified membranes.

### 3.2. Effects of Transmembrane Pressure on Filtration Properties and Purification Efficiency

There are numerous publications that report that during the membrane filtration of oily contaminants, the flux increase that can be achieved by using higher transmembrane pressures is very limited. This is due to the formation of a more compact hydrophobic cake layer and the occurrence of an even more significant pore fouling resulting from the more deformable oil droplets at higher pressures [31,36,42]. Due to this, the possible transmembrane pressure-dependent beneficial effect of the TiO_2_/CNT_2%_ coating was also investigated. The filtration of the oil emulsion was carried out at 0.1, 0.2, and 0.3 MPa transmembrane pressures with both the commercial PVDF membrane and the TiO_2_/CNT_2%_-coated membrane. During these experiments, fluxes and purification efficiencies were measured, and filtration resistances were calculated.

#### 3.2.1. Effect of the Transmembrane Pressure on Fluxes and Filtration Resistances

**Figure 5:**
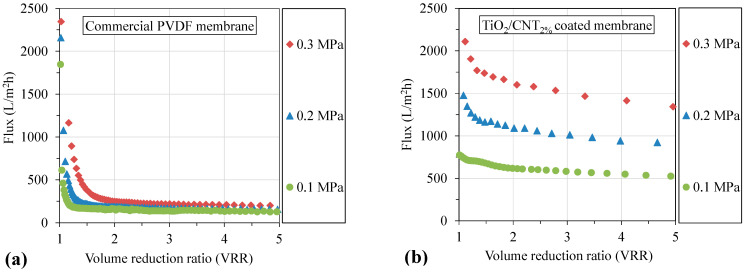
Effects of the transmembrane pressure on fluxes measured during the filtration of oil emulsions with the commercial membrane (**a**) and the TiO_2_/CNT_2%_ nanocomposite-modified membrane (**b**).

In the case of the uncoated commercial PVDF membrane, the flux (Figure 5a) was just slightly increased by increasing the transmembrane pressure: 130, 155, and 190 L/m^2^h fluxes were measured at VRR = 5, when 0.1, 0.2, and 0.3 MPa pressures were applied, respectively. However, for the TiO_2_/CNT_2%_ nanocomposite-coated PVDF membrane, the fluxes (Figure 5b) increased significantly with the increasing transmembrane pressure: 510, 900, and 1340 L/m^2^h fluxes were measured at 0.1, 0.2, and 0.3 MPa pressures, respectively.

For the uncoated PVDF membrane, the fluxes decreased intensely from the very beginning of the filtrations despite the relatively high initial values. At a VRR of 2, the fluxes were already lower than 160, 190, and 250 L/m^2^h, when 0.1, 0.2, and 0.3 MPa pressures were applied, respectively. The results demonstrate the rapid build-up of a hydrophobic oily cake layer on the uncoated membrane and suggest a more intense fouling at higher transmembrane pressures.

For the TiO_2_/CNT_2%_ composite-coated membrane, the fluxes were significantly higher, not just at the beginning of the experiments, but they were stabilized at high values. Until the end of the filtration experiments, the fluxes remained close to two and three times higher at pressures of 0.2 and 0.3 MPa, compared to the flux obtained at a 0.1 MPa pressure. Comparing the values of the commercial and TiO_2_/CNT_2%_-coated membranes, 4–7 times higher fluxes (510, 900, and 1340 L/m^2^h) were achieved with the modified membranes, depending on the applied transmembrane pressure (0.1, 0.2, and 0.3 MPa, respectively). To emphasize the importance of this beneficial property, we compared the results with another recent study [40], in which oil emulsions were filtered with PVDF−MWCNT membranes at four different transmembrane pressures of 0.1, 0.2, 0.3, and 0.4 MPa. The fluxes were between ~380 and 665 L/m^2^h at a 0.1 MPa transmembrane pressure, depending on the amount and the surface nature of the used MWCNTs. Despite the four times higher transmembrane pressure, the flux enhancement remained <2.5% in all cases, and the highest flux was only ~675 L/m^2^h at a 0.4 MPa transmembrane pressure. Furthermore, in an earlier study, 265 and 350 L/m^2^h fluxes were measured at 0.12 and 0.24 MPa transmembrane pressures (for TiO_2_-modified ceramic membranes), which means that only a 32% flux enhancement was achieved at a double pressure [19]. 

For a further comparison of the presented results with some other relevant studies (which also investigated the membrane filtration of oil emulsions with TiO_2_- and/or CNT-modified membranes), the most important experimental parameters (transmembrane pressure, type of the membrane, and used oily contaminant), fluxes, flux recovery ratios, and purification efficiencies were summarized in Table 1.

**Figure 6:**
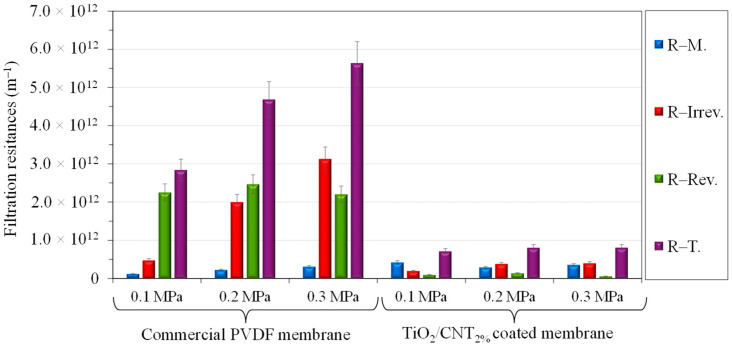
Effects of the transmembrane pressure on the filtration resistance for the commercial and TiO_2_/CNT_2%_ nanocomposite-modified membranes.

Different filtration resistances were calculated (as detailed in Section 2.5) in this series as well (Figure 6). The total filtration resistance increased significantly by higher transmembrane pressures in the case of the unmodified commercial PVDF membrane. However, for the nanocomposite-modified membrane, only a slight growth was observed.

On the one hand, these results demonstrate that higher pressures caused not only the rapid build-up of a potentially more compact hydrophobic cake layer on the commercial PVDF membrane, but also resulted in more permanently adhered contaminants and/or a more intense fouling of the pores, and therefore significantly increased irreversible resistances, which resulted in very low flux recovery ratios (20%, 10%, and 9% at 0.1, 0.2, and 0.3 MPa pressures, respectively). On the other hand, the composite coating was successfully applied to reduce the adhesion of the oil droplets at all three investigated transmembrane pressures. Therefore, not only were the fluxes outstandingly high, but much higher flux recovery ratios were obtained by modifying the membranes with the TiO_2_/CNT_2%_ nanocomposite: FRRs were 68%, 43%, and 47% at 0.1, 0.2, and 0.3 MPa pressures, respectively.

#### 3.2.2. Effect of the Transmembrane Pressure on the Purification Efficiency

**Figure 7:**
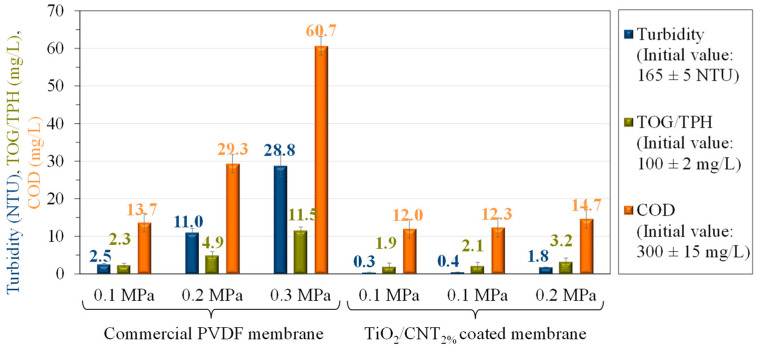
The turbidity, the extractable oil content (TOG/TPH), and the chemical oxygen demand (COD) of the permeates for different membranes at different transmembrane pressures.

The measured turbidity, TOG/TPH, and COD values of the permeates produced with the different membranes at different transmembrane pressures are presented in Figure 7.

Based on the contaminant content of the permeates obtained at a 0.1 MPa transmembrane pressure, the TiO_2_/CNT_2%_ composite coating affected the purification efficiency beneficially, since lower contamination values were measured using all three analytical methods (the turbidity was reduced from 2.5 to 0.3 NTU due to the membrane surface modification).

Another remarkable result was that higher transmembrane pressures resulted in significantly more contaminated permeates in the case of the commercial membrane. At the same time, for the TiO_2_/CNT_2%_-coated membrane, relatively low values were measured even at higher transmembrane pressures. For example, the turbidity of the permeate was ~16 times higher for the unmodified membrane than for the TiO_2_/CNT_2%_-coated membrane at a 0.3 MPa transmembrane pressure.

The achieved purification efficiencies can be summarized as follows. Depending on the applied transmembrane pressure (0.1/0.2/0.3 MPa), for the commercial membrane, 98.5%/93.3%/82.5%, 95.4%/90.2%/79.8%, and 97.7%/95.1%/88.5% purification efficiencies were calculated regarding the turbidity, COD, and TOG/TPH values, respectively. For the TiO_2_/CNT_2%_-coated membrane, 99.8%/99.7%/98.9%, 96.0%/95.9%/95.1%, and 98.1%/97.9%/96.8% purification efficiencies were measured (regarding the turbidity, COD, and TOG/TPH values, respectively), which represent good rejections in comparison with other studies (Table 1). These results undoubtedly prove the beneficial effect of the applied membrane surface modification in terms of the purification efficiency, which becomes even more significant at higher transmembrane pressures.

### 3.3. Future Research Directions

Based on the results and discussion presented above, the following aspects could be worth investigating:

- Utilization of TiO_2_/CNT composites to modify membranes via other methods including blending and grafting immobilization techniques. This could result in the more durable immobilization of nanomaterials for long-term applications [11,16].
- Investigation of possibilities with nanocomposites containing TiO_2_ and –OH and/or –COOH-functionalized (oxidized) CNTs, as the functionalization may ensure even more negative zeta potentials and reduced hydrophobicity of CNTs [31].
- Exploration of the achievable advantages and limitations of these nanocomposite-based membrane modifications in terms of the properties of the water matrix (pH, ion content, etc.) since these are also significant influencing factors [43].

## 4. Conclusions

TiO_2_/CNT nanocomposite-based surface modification of PVDF membranes was ef-fective to increase the flux during the membrane filtration of oil emulsions.

For the TiO_2_/CNT_1%_-modified membrane ~2.5 times higher fluxes (310 L/m^2^h) and for TiO_2_/CNT_2%_ ~4 times higher fluxes (510 L/m^2^h) were measured (at a 0.1 MPa transmembrane pressure) compared to that for the unmodified membrane (130 L/m^2^h). Modification with TiO_2_/CNT_5%_ and pure TiO_2_ resulted in very similar fluxes for these two membranes: 170 L/m^2^h and 185 L/m^2^h, respectively. The lowest total, reversible, and irreversible filtration resistances were obtained for the membrane modified with TiO_2_/CNT_2%_.

It was demonstrated that for the commercial PVDF membrane, the flux could not be significantly increased by using higher transmembrane pressures and the purification efficiency decreased significantly (down to 82.5%, 79.8%, and 88.5%, regarding the turbidity, the COD, and the TOG/TPH, respectively) by applying higher pressures. However, the TiO_2_/CNT_2%_-coated membrane provided ~6–7 times higher fluxes (compared to the unmodified membrane) at 0.2 and 0.3 MPa transmembrane pressures (900 L/m^2^h and 1340 L/m^2^h, respectively) and excellent purification efficiencies (95.1–98.9%) at the highest pressure as well.

In summary, TiO_2_/CNT_2%_ nanocomposites are promising for modifying membranes used to filtrate oily wastewaters to achieve outstanding flux, cleanability, and purification efficiency. The beneficial effects are even more significant at higher transmembrane pressures, which is an undoubtedly important advantage regarding industrial applications.

## Figures and Tables

**Table 1:** Comparison of different TiO_2_- and/or CNT-modified membranes (used for the filtration of oil emulsions) with the current study.

Flux (L/m^2^h)	Pressure	Purification Efficiency (%)	Membrane	Oil Content of the Emulsion	Flux Recovery Ratio (%)	Ref.
245–350	0.12–0.24 MPa	–	Ceramic−TiO_2_	hydraulic oil (concentration is missing)	not investigated	[19], 2014
30–70	vacuum	80–99.7	PVDF−TiO_2_	250 mg/L (cutting oil)	not investigated	[20], 2015
380–675	0.1–0.4 MPa	~96–99.8	PVDF−MWCNT	100 mg/L oil (synthesized refinery wastewater)	not investigated	[31], 2015
382	0.09 MPa	~99	PVDF−TiO_2_	~10,000 mg/L diesel oil	not investigated	[22], 2016
301–362	0.1 MPa	95–99	PVDF−TiO_2_/MWCNT, PVDF−TiO_2_	28 mg/L oil (industrial oil-contaminated wastewater)	64–77 (after water cleaning)	[27], 2020
43–55	0.05 MPa	83–94	* NWF−TiO_2_ (* nonwoven fabric)	10,000 mg/L (crude oil)	~20–50 (after water, 1 g/L NaClO, and UV cleaning)	[18], 2022
~30–100	0.2 MPa	>99.5	PVDF−MWCNT−polypyrrole	500 mg/L (crude oil)	80–90 (after 0.1 M NaOH cleaning)	[40], 2022
510–1340	0.1–0.3 MPa	95.1–99.8	PVDF−TiO_2_/MWCNT	100 mg/L (crude oil)	47–68 (after water cleaning)	This study

## Data Availability

Data are contained within the article.
